# 4-[Bis(4-fluoro­phen­yl)meth­yl]-1-[(2*E*)-3-phenyl­prop-2-en-1-yl]piperazin-1-ium 3-carb­oxy­propano­ate

**DOI:** 10.1107/S1600536813000706

**Published:** 2013-01-19

**Authors:** Channappa N. Kavitha, Hemmige S. Yathirajan, Badiadka Narayana, Thomas Gerber, Benjamin van Brecht, Richard Betz

**Affiliations:** a University of Mysore, Department of Studies in Chemistry, Manasagangotri, Mysore 570 006, India; b Mangalore University, Department of Studies in Chemistry, Mangalagangotri 574 199, India; c Nelson Mandela Metropolitan University, Summerstrand Campus, Department of Chemistry, University Way, Summerstrand, PO Box 77000, Port Elizabeth, 6031, South Africa

## Abstract

In the title salt, C_26_H_27_F_2_N_2_^+^·C_4_H_5_O_4_^−^, the piperazine N atom bearing the vinylic substituent is protonated. The piperazine ring adopts a chair conformation. In ther crystal, the succinate monoanions are connected *via* short O—H⋯O hydrogen bonds between the carb­oxy­lic acid and carboxyl­ate groups into undulating chains extending along [001] and the flunarizinium monocations are attached to these chains *via* N^+^—H⋯O^−^ hydrogen bonds. C—H⋯O inter­actions connect these chains into a three-dimensional network. The shortest centroid–centroid distance of 3.7256 (10) Å was found between one of the fluorinated benzene rings and the non-fluorinated phenyl ring in the neighbouring mol­ecule related by a glide plane.

## Related literature

For pharmaceutical properties of flunarizine, see: Holmes *et al.* (1984 ▶); Amery (1983 ▶) and of piperazine derivatives, see: Brockunier *et al.* (2004 ▶); Bogatcheva *et al.* (2006 ▶); Elliott (2011 ▶). For related structures, see: Betz *et al.* (2011*a* ▶,*b* ▶); Dayananda *et al.* (2012*a* ▶,*b* ▶); Fillers & Hawkinson (1982 ▶); Vanier & Brisse (1983 ▶). For puckering analysis of six-membered rings, see: Cremer & Pople (1975 ▶); Boeyens (1978 ▶). For graph-set analysis of hydrogen bonds, see: Etter *et al.* (1990 ▶); Bernstein *et al.* (1995 ▶).
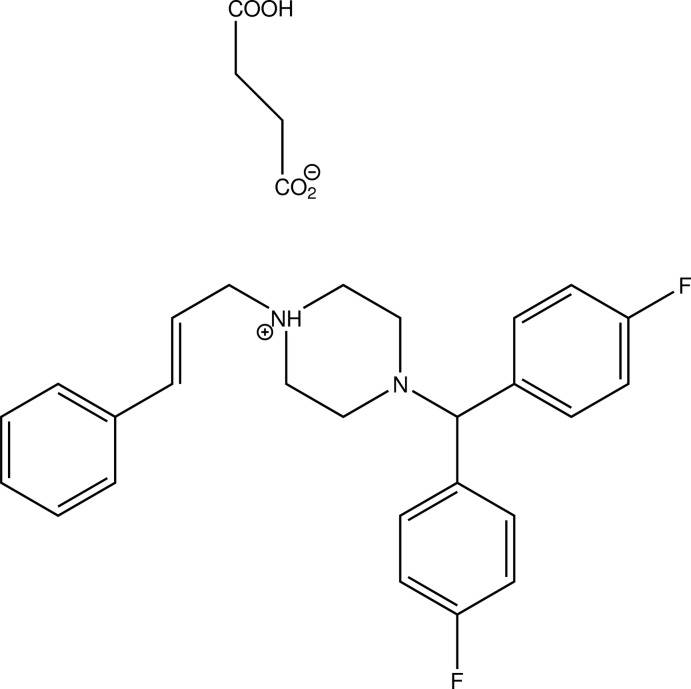


## Experimental

### 

#### Crystal data

C_26_H_27_F_2_N_2_^+^·C_4_H_5_O_4_^−^
*M* *_r_* = 522.58
Monoclinic, 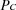

*a* = 10.7824 (2) Å
*b* = 10.6270 (2) Å
*c* = 11.2364 (2) Å
β = 91.678 (1)°
*V* = 1286.97 (4) Å^3^
*Z* = 2
Mo *K*α radiation
μ = 0.10 mm^−1^
*T* = 200 K
0.56 × 0.29 × 0.16 mm

#### Data collection

Bruker APEXII CCD diffractometer
Absorption correction: multi-scan (*SADABS*; Bruker, 2008 ▶) *T*_min_ = 0.947, *T*_max_ = 0.985
16870 measured reflections
3619 independent reflections
3524 reflections with *I* > 2σ(*I*)
*R*_int_ = 0.014

#### Refinement

*R*[*F* ^2^ > 2σ(*F* ^2^)] = 0.029
*wR*(*F* ^2^) = 0.081
*S* = 1.03
3619 reflections
348 parameters
2 restraints
H atoms treated by a mixture of independent and constrained refinement
Δρ_max_ = 0.25 e Å^−3^
Δρ_min_ = −0.15 e Å^−3^

### 

Data collection: *APEX2* (Bruker, 2010 ▶); cell refinement: *SAINT* (Bruker, 2010 ▶); data reduction: *SAINT*; program(s) used to solve structure: *SHELXS97* (Sheldrick, 2008 ▶); program(s) used to refine structure: *SHELXL97* (Sheldrick, 2008 ▶); molecular graphics: *ORTEP-3* (Farrugia, 2012 ▶) and *Mercury* (Macrae *et al.*, 2008 ▶); software used to prepare material for publication: *SHELXL97* and *PLATON* (Spek, 2009 ▶).

## Supplementary Material

Crystal structure: contains datablock(s) I, global. DOI: 10.1107/S1600536813000706/gk2534sup1.cif

Supplementary material file. DOI: 10.1107/S1600536813000706/gk2534Isup2.cdx

Structure factors: contains datablock(s) I. DOI: 10.1107/S1600536813000706/gk2534Isup3.hkl

Supplementary material file. DOI: 10.1107/S1600536813000706/gk2534Isup4.cml

Additional supplementary materials:  crystallographic information; 3D view; checkCIF report

## Figures and Tables

**Table 1:** Hydrogen-bond geometry (Å, °)

*D*—H⋯*A*	*D*—H	H⋯*A*	*D*⋯*A*	*D*—H⋯*A*
N2—H72⋯O1	0.96 (2)	1.73 (2)	2.6795 (16)	174 (2)
O3—H3⋯O1^i^	0.84	1.84	2.6564 (16)	162
C4—H4*A*⋯O4^ii^	0.99	2.58	3.287 (3)	128
C4—H4*B*⋯O2^iii^	0.99	2.39	3.3167 (19)	155
C25—H25⋯O2^iv^	0.95	2.53	3.4168 (19)	155
C12—H12⋯*Cg* ^v^	0.95	2.81	3.7511 (17)	170

Symmetry codes: (i) 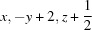; (ii) 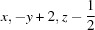; (iii) 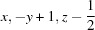; (iv) 
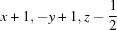
; (v) 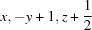.

**Fig. 1.**
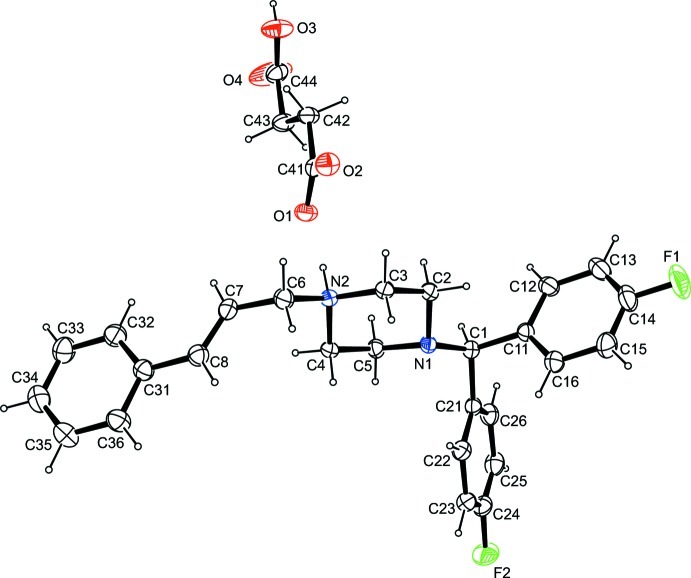
The molecular structure of the title compound, with atom labels and anisotropic displacement ellipsoids drawn at 50% probability level.

**Fig. 2.**
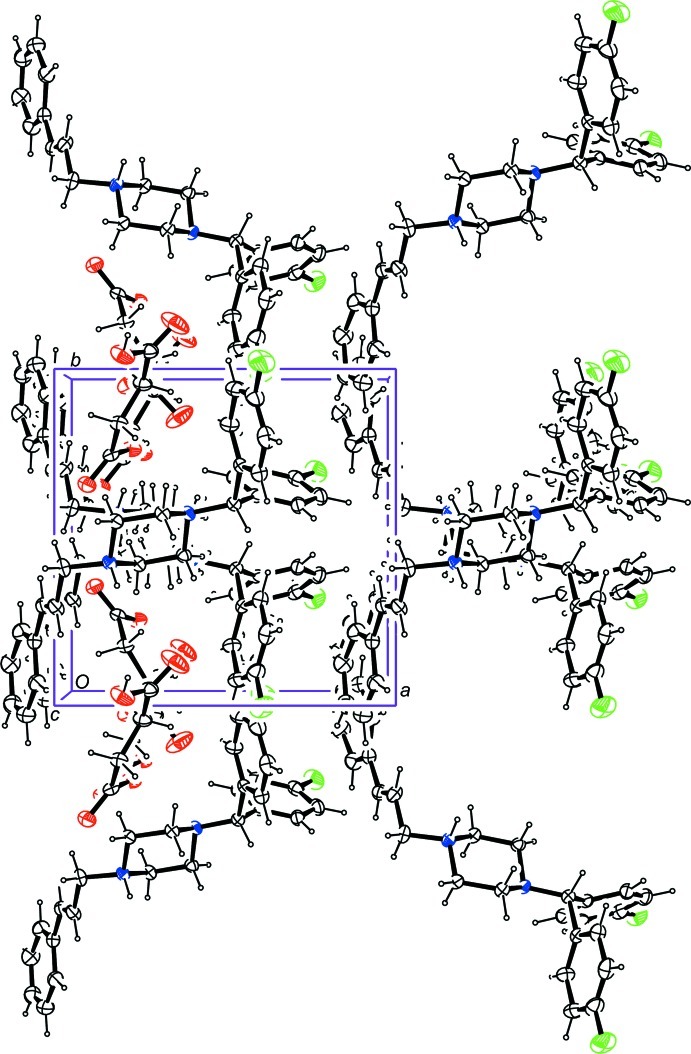
Molecular packing of the title compound, viewed along [0 0 1] (anisotropic displacement ellipsoids drawn at the 50% probability level).

## supplementary crystallographic information

### Comment

Flunarizine is a drug classified as a calcium channel blocker (Amery, 1983). A review of its pharmacodynamic and pharmacokinetic properties and therapeutic use has been published (Holmes *et al.*, 1984). Piperazines are among the most important building blocks in today's drug discovery and are found in biologically active compounds in a number of different therapeutic areas (Brockunier *et al.*, 2004; Bogatcheva *et al.*, 2006), and a review about the current pharmacological and toxicological information for piperazine derivatives is available (Elliott, 2011). The crystal structures of several related compounds are apparent in the literature (Fillers & Hawkinson, 1982; Vanier & Brisse, 1983; Betz *et al.*, 2011*a*,*b*; Dayananda *et al.*, 2012*a*,*b*). In continuation of our research about the salts of pharmacologically active compounds the title compound was synthesized and its crystal structure was determined.

Protonation of the flunarizine scaffold occurred on the nitrogen atom bearing the vinylic substituent. According to a puckering analysis (Cremer & Pople, 1975; Boeyens, 1978), the central 1,4-diazacyclohexane ring adopts a ^4^*C*_1_ conformation with both nitrogen atoms acting as the flap atoms (^N2^*C*_N1_). The C=C bond in the vinylic substituent is (*E*)-configured. The least-squares planes defined by the individual carbon atoms of the fluorinated phenyl moieties enclose an angle of 78.09 (8) °. The plane defined by the carbon atoms of the non-halogenated phenyl ring intersects at angles of 11.08 (8) ° and 87.51 (8) ° with the two aforementioned planes. The succinate monoanion is essentially flat (r.m.s. of all fitted non-hydrogen atoms = 0.0955 Å) with one of the carbon atoms of a methylene group deviating most from the common plane by 0.160 (1) Å (Fig. 1). The succinate monoanion adopts a zigzag conformation.

In the crystal, C–H···O contacts whose range falls by up to more than 0.3 Å below the sum of van-der-Waals radii of the atoms participating are observed next to classical hydrogen bonds of the O–H···O and N–H···O type. The C–H···O contacts are supported by both hydrogen atoms of an intracyclic methylene group bonded to the protonated nitrogen atom as well as one hydrogen atom in *ortho*-position to a fluorine atom in one of the fluorophenyl moieties as donors. The protonated carboxyl group forms a hydrogen bond to the deprotonated carboxyl group, the latter one also serving as acceptor for the N–H···O type hydrogen bonds. In addition, a C–H···π interaction involving one of the hydrogen atoms in *meta*-position to the fluorine atoms on the fluorophenyl moiety that does not contribute to the C–H···O contacts as described above as the donor and the aromtic system of the non-halogenated phenyl ring as the acceptor is apparent. Metrical parameters as well as information about the symmetry of these contacts are summarized in Table 1. In total, the entities of the title compound are connected to a three-dimensional network. In terms of graph-set analysis (Etter *et al.*, 1990; Bernstein *et al.*, 1995), the descriptor for these contacts is *DC*^1^_1_(7) for the classical hydrogen bonds on the unary level. The C–H···O contacts necessitate a *DDD* descriptor on the same level. The shortest intercentroid distance between twomoiety aromatic systems was found at 3.7256 (10) Å and is apparent between one of the fluorinated and the non-halogenated phenyl moiety in neighbouring cations.

The packing of the title compound in the crystal structure is shown in Figure 2.

### Experimental

Flunarizine (4.05 g, 0.01 mol) and succinic acid (1.18 g, 0.01 mol) were dissolved in hot *N*,*N*-dimethylformamide and reacted for 30 minutes. The resulting solution was allowed to cool slowly at room temperature upon which crystals of the title compound appeared in the course of several days. The latter were of sufficient quality for the X-ray diffraction studies.

### Refinement

Carbon-bound H atoms were placed in calculated positions (C–H 0.95 Å for aromatic and vinylic carbon atoms, C–H 0.99 Å for methylene groups and C–H 1.00 Å for the methine group) and were included in the refinement in the riding model approximation, with *U*(H) set to 1.2*U*_eq_(C). The H atom of the hydroxyl group was allowed to rotate with a fixed angle around the C–O bond to best fit the experimental electron density [HFIX 147 in the *SHELX* program suite (Sheldrick, 2008)] with *U*(H) set to 1.5*U*_eq_(O). The nitrogen-bound H atom was located on a difference Fourier map and refined freely.

### Crystal data

**Table:**

C_26_H_27_F_2_N_2_^+^·C_4_H_5_O_4_^−^	*F*(000) = 552
*M**_r_* = 522.58	*D*_x_ = 1.349 Mg m^−^^3^
Monoclinic, *P**c*	Melting point = 383–378 K
Hall symbol: P -2yc	Mo *K*α radiation, λ = 0.71073 Å
*a* = 10.7824 (2) Å	Cell parameters from 9972 reflections
*b* = 10.6270 (2) Å	θ = 2.6–29.6°
*c* = 11.2364 (2) Å	µ = 0.10 mm^−^^1^
β = 91.678 (1)°	*T* = 200 K
*V* = 1286.97 (4) Å^3^	Rectangular, yellow
*Z* = 2	0.56 × 0.29 × 0.16 mm

### Data collection

**Table:**

Bruker APEXII CCD diffractometer	3619 independent reflections
Radiation source: fine-focus sealed tube	3524 reflections with *I* > 2σ(*I*)
Graphite monochromator	*R*_int_ = 0.014
φ and ω scans	θ_max_ = 29.6°, θ_min_ = 2.6°
Absorption correction: multi-scan (*SADABS*; Bruker, 2008)	*h* = −14→14
*T*_min_ = 0.947, *T*_max_ = 0.985	*k* = −14→14
16870 measured reflections	*l* = −15→15

### Refinement

**Table:**

Refinement on *F*^2^	Primary atom site location: structure-invariant direct methods
Least-squares matrix: full	Secondary atom site location: difference Fourier map
*R*[*F*^2^ > 2σ(*F*^2^)] = 0.029	Hydrogen site location: inferred from neighbouring sites
*wR*(*F*^2^) = 0.081	H atoms treated by a mixture of independent and constrained refinement
*S* = 1.03	*w* = 1/[σ^2^(*F*_o_^2^) + (0.0532*P*)^2^ + 0.1548*P*] where *P* = (*F*_o_^2^ + 2*F*_c_^2^)/3
3619 reflections	(Δ/σ)_max_ < 0.001
348 parameters	Δρ_max_ = 0.25 e Å^−^^3^
2 restraints	Δρ_min_ = −0.15 e Å^−^^3^

### Special details

**Table:**

Refinement. Due to the absence of a strong anomalous scatterer, the Flack parameter is meaningless. Thus, Friedel opposites (3016 pairs) have been merged and the item was removed from the CIF.

### Fractional atomic coordinates and isotropic or equivalent isotropic displacement parameters (Å^2^)

**Table:**

	*x*	*y*	*z*	*U*_iso_*/*U*_eq_	
F1	0.60945 (15)	−0.00547 (14)	0.96672 (13)	0.0575 (3)	
F2	0.76769 (12)	0.30206 (12)	0.22338 (10)	0.0453 (3)	
N1	0.39495 (10)	0.42901 (11)	0.63218 (10)	0.0199 (2)	
N2	0.16828 (10)	0.57409 (11)	0.64797 (10)	0.0205 (2)	
H72	0.192 (2)	0.640 (2)	0.7023 (19)	0.028 (5)*	
C1	0.52807 (12)	0.40104 (13)	0.65297 (11)	0.0204 (2)	
H1	0.5681	0.4768	0.6903	0.024*	
C2	0.32435 (13)	0.43027 (13)	0.74174 (12)	0.0223 (2)	
H2A	0.3558	0.4980	0.7950	0.027*	
H2B	0.3351	0.3490	0.7839	0.027*	
C3	0.18784 (13)	0.45191 (13)	0.71164 (12)	0.0222 (2)	
H3A	0.1560	0.3822	0.6609	0.027*	
H3B	0.1406	0.4521	0.7859	0.027*	
C4	0.24729 (12)	0.57850 (13)	0.54108 (11)	0.0214 (2)	
H4A	0.2400	0.6623	0.5031	0.026*	
H4B	0.2181	0.5146	0.4825	0.026*	
C5	0.38119 (13)	0.55330 (13)	0.57621 (12)	0.0220 (2)	
H5A	0.4325	0.5572	0.5047	0.026*	
H5B	0.4111	0.6190	0.6325	0.026*	
C6	0.03255 (13)	0.58877 (15)	0.61516 (13)	0.0268 (3)	
H6A	0.0047	0.5155	0.5669	0.032*	
H6B	−0.0159	0.5900	0.6886	0.032*	
C7	0.00768 (14)	0.70704 (15)	0.54620 (14)	0.0282 (3)	
H7	0.0228	0.7860	0.5836	0.034*	
C8	−0.03506 (14)	0.70463 (14)	0.43376 (14)	0.0268 (3)	
H8	−0.0467	0.6237	0.3993	0.032*	
C11	0.54868 (12)	0.29049 (13)	0.73680 (12)	0.0216 (2)	
C12	0.60202 (14)	0.31075 (15)	0.84953 (13)	0.0274 (3)	
H12	0.6246	0.3936	0.8733	0.033*	
C13	0.62266 (16)	0.21099 (19)	0.92779 (14)	0.0351 (3)	
H13	0.6591	0.2248	1.0047	0.042*	
C14	0.58933 (17)	0.09282 (18)	0.89143 (16)	0.0365 (4)	
C15	0.53618 (18)	0.06793 (16)	0.78097 (16)	0.0364 (3)	
H15	0.5139	−0.0154	0.7584	0.044*	
C16	0.51610 (15)	0.16833 (14)	0.70341 (14)	0.0291 (3)	
H16	0.4797	0.1534	0.6267	0.035*	
C21	0.58957 (12)	0.37651 (12)	0.53494 (12)	0.0212 (2)	
C22	0.52566 (13)	0.32091 (13)	0.43931 (13)	0.0243 (2)	
H22	0.4402	0.3010	0.4460	0.029*	
C23	0.58533 (15)	0.29394 (14)	0.33373 (13)	0.0279 (3)	
H23	0.5418	0.2559	0.2684	0.033*	
C24	0.70925 (15)	0.32415 (15)	0.32721 (14)	0.0301 (3)	
C25	0.77684 (14)	0.37818 (16)	0.41996 (15)	0.0317 (3)	
H25	0.8626	0.3964	0.4132	0.038*	
C26	0.71468 (13)	0.40504 (15)	0.52386 (13)	0.0269 (3)	
H26	0.7586	0.4437	0.5886	0.032*	
C31	−0.06628 (13)	0.81225 (14)	0.35691 (13)	0.0257 (3)	
C32	−0.05674 (15)	0.93704 (15)	0.39524 (15)	0.0310 (3)	
H32	−0.0249	0.9550	0.4731	0.037*	
C33	−0.09348 (17)	1.03489 (17)	0.32034 (17)	0.0369 (3)	
H33	−0.0861	1.1194	0.3471	0.044*	
C34	−0.14092 (17)	1.00983 (18)	0.20662 (17)	0.0379 (4)	
H34	−0.1675	1.0769	0.1562	0.046*	
C35	−0.14940 (18)	0.88675 (19)	0.16704 (15)	0.0378 (4)	
H35	−0.1810	0.8693	0.0890	0.045*	
C36	−0.11186 (16)	0.78902 (16)	0.24134 (14)	0.0326 (3)	
H36	−0.1172	0.7048	0.2132	0.039*	
O1	0.21803 (11)	0.76463 (10)	0.79808 (9)	0.0295 (2)	
O2	0.09155 (11)	0.65696 (11)	0.91333 (10)	0.0310 (2)	
O3	0.20616 (15)	1.03647 (13)	1.15502 (12)	0.0423 (3)	
H3	0.2229	1.0913	1.2069	0.063*	
O4	0.35531 (19)	1.13282 (17)	1.06159 (18)	0.0694 (6)	
C41	0.16219 (13)	0.74667 (13)	0.89574 (12)	0.0226 (2)	
C42	0.18904 (14)	0.83825 (13)	0.99780 (12)	0.0254 (3)	
H42A	0.1093	0.8623	1.0328	0.030*	
H42B	0.2387	0.7939	1.0602	0.030*	
C43	0.25742 (15)	0.95766 (14)	0.96455 (13)	0.0279 (3)	
H43A	0.3386	0.9337	0.9325	0.034*	
H43B	0.2094	1.0003	0.8999	0.034*	
C44	0.27975 (16)	1.05070 (14)	1.06472 (14)	0.0296 (3)	

### Atomic displacement parameters (Å^2^)

**Table:**

	*U*^11^	*U*^22^	*U*^33^	*U*^12^	*U*^13^	*U*^23^
F1	0.0687 (8)	0.0515 (7)	0.0524 (7)	0.0115 (6)	0.0015 (6)	0.0322 (6)
F2	0.0511 (6)	0.0496 (6)	0.0364 (5)	0.0085 (5)	0.0228 (5)	0.0009 (5)
N1	0.0200 (5)	0.0214 (5)	0.0185 (5)	0.0035 (4)	0.0028 (4)	0.0027 (4)
N2	0.0216 (5)	0.0220 (5)	0.0178 (5)	0.0036 (4)	0.0005 (4)	−0.0015 (4)
C1	0.0204 (5)	0.0204 (5)	0.0203 (5)	0.0017 (4)	0.0001 (4)	0.0002 (4)
C2	0.0239 (6)	0.0246 (6)	0.0184 (5)	0.0038 (5)	0.0023 (4)	0.0026 (4)
C3	0.0246 (6)	0.0226 (6)	0.0197 (5)	0.0025 (5)	0.0035 (4)	0.0017 (4)
C4	0.0234 (6)	0.0248 (6)	0.0160 (5)	0.0035 (5)	0.0021 (4)	0.0011 (4)
C5	0.0228 (6)	0.0212 (6)	0.0221 (6)	0.0020 (5)	0.0017 (4)	0.0033 (4)
C6	0.0204 (6)	0.0346 (7)	0.0253 (6)	0.0044 (5)	0.0001 (5)	0.0004 (5)
C7	0.0267 (6)	0.0290 (7)	0.0288 (7)	0.0081 (5)	−0.0017 (5)	−0.0033 (5)
C8	0.0254 (6)	0.0271 (6)	0.0277 (6)	0.0009 (5)	−0.0005 (5)	−0.0010 (5)
C11	0.0207 (5)	0.0235 (6)	0.0205 (6)	0.0036 (5)	0.0010 (4)	0.0019 (5)
C12	0.0264 (6)	0.0332 (7)	0.0226 (6)	0.0017 (5)	−0.0002 (5)	0.0007 (5)
C13	0.0315 (7)	0.0496 (9)	0.0242 (7)	0.0048 (7)	−0.0009 (5)	0.0089 (6)
C14	0.0359 (8)	0.0392 (8)	0.0348 (8)	0.0085 (7)	0.0061 (6)	0.0178 (7)
C15	0.0461 (9)	0.0245 (7)	0.0388 (8)	0.0033 (6)	0.0047 (7)	0.0073 (6)
C16	0.0361 (7)	0.0233 (6)	0.0278 (7)	0.0015 (5)	−0.0005 (5)	0.0017 (5)
C21	0.0223 (6)	0.0198 (6)	0.0215 (5)	0.0031 (4)	0.0027 (4)	0.0029 (4)
C22	0.0238 (6)	0.0236 (6)	0.0257 (6)	0.0004 (5)	0.0018 (5)	0.0004 (5)
C23	0.0353 (7)	0.0244 (6)	0.0240 (6)	0.0043 (5)	0.0027 (5)	−0.0003 (5)
C24	0.0353 (8)	0.0272 (7)	0.0285 (7)	0.0082 (6)	0.0118 (6)	0.0054 (5)
C25	0.0251 (6)	0.0320 (8)	0.0383 (8)	0.0043 (5)	0.0082 (6)	0.0072 (6)
C26	0.0221 (6)	0.0281 (7)	0.0304 (7)	0.0012 (5)	0.0006 (5)	0.0041 (5)
C31	0.0217 (6)	0.0297 (7)	0.0258 (6)	−0.0004 (5)	0.0000 (5)	0.0006 (5)
C32	0.0303 (7)	0.0314 (7)	0.0311 (7)	−0.0006 (6)	−0.0012 (6)	−0.0020 (6)
C33	0.0388 (8)	0.0285 (7)	0.0434 (9)	0.0013 (6)	−0.0005 (7)	0.0014 (6)
C34	0.0354 (8)	0.0396 (9)	0.0387 (8)	0.0021 (7)	−0.0011 (6)	0.0096 (7)
C35	0.0412 (8)	0.0432 (9)	0.0286 (7)	−0.0014 (7)	−0.0055 (6)	0.0041 (6)
C36	0.0358 (8)	0.0339 (8)	0.0280 (7)	−0.0027 (6)	−0.0029 (6)	−0.0013 (6)
O1	0.0443 (6)	0.0237 (5)	0.0207 (4)	−0.0016 (4)	0.0052 (4)	−0.0010 (4)
O2	0.0343 (5)	0.0262 (5)	0.0328 (5)	−0.0049 (4)	0.0033 (4)	−0.0012 (4)
O3	0.0591 (8)	0.0372 (6)	0.0314 (6)	−0.0147 (6)	0.0143 (5)	−0.0140 (5)
O4	0.0814 (12)	0.0525 (9)	0.0770 (11)	−0.0382 (9)	0.0448 (10)	−0.0354 (9)
C41	0.0273 (6)	0.0185 (5)	0.0219 (5)	0.0044 (5)	−0.0009 (4)	0.0002 (4)
C42	0.0345 (7)	0.0219 (6)	0.0200 (5)	−0.0021 (5)	0.0049 (5)	−0.0017 (4)
C43	0.0377 (7)	0.0217 (6)	0.0248 (6)	−0.0033 (5)	0.0075 (5)	−0.0025 (5)
C44	0.0356 (7)	0.0221 (6)	0.0312 (7)	−0.0006 (5)	0.0061 (6)	−0.0041 (5)

### Geometric parameters (Å, º)

**Table:**

F1—C14	1.3576 (18)	C15—C16	1.391 (2)
F2—C24	1.3625 (17)	C15—H15	0.9500
N1—C2	1.4663 (16)	C16—H16	0.9500
N1—C5	1.4686 (17)	C21—C22	1.3911 (19)
N1—C1	1.4776 (16)	C21—C26	1.3918 (19)
N2—C4	1.4934 (16)	C22—C23	1.3957 (19)
N2—C3	1.4944 (17)	C22—H22	0.9500
N2—C6	1.5069 (17)	C23—C24	1.378 (2)
N2—H72	0.96 (2)	C23—H23	0.9500
C1—C11	1.5181 (18)	C24—C25	1.379 (2)
C1—C21	1.5225 (18)	C25—C26	1.393 (2)
C1—H1	1.0000	C25—H25	0.9500
C2—C3	1.5182 (19)	C26—H26	0.9500
C2—H2A	0.9900	C31—C36	1.397 (2)
C2—H2B	0.9900	C31—C32	1.397 (2)
C3—H3A	0.9900	C32—C33	1.388 (2)
C3—H3B	0.9900	C32—H32	0.9500
C4—C5	1.5094 (19)	C33—C34	1.388 (3)
C4—H4A	0.9900	C33—H33	0.9500
C4—H4B	0.9900	C34—C35	1.384 (3)
C5—H5A	0.9900	C34—H34	0.9500
C5—H5B	0.9900	C35—C36	1.385 (2)
C6—C7	1.497 (2)	C35—H35	0.9500
C6—H6A	0.9900	C36—H36	0.9500
C6—H6B	0.9900	O1—C41	1.2813 (17)
C7—C8	1.332 (2)	O2—C41	1.2398 (19)
C7—H7	0.9500	O3—C44	1.3150 (19)
C8—C31	1.466 (2)	O3—H3	0.8400
C8—H8	0.9500	O4—C44	1.195 (2)
C11—C12	1.3924 (19)	C41—C42	1.5254 (19)
C11—C16	1.394 (2)	C42—C43	1.520 (2)
C12—C13	1.391 (2)	C42—H42A	0.9900
C12—H12	0.9500	C42—H42B	0.9900
C13—C14	1.366 (3)	C43—C44	1.512 (2)
C13—H13	0.9500	C43—H43A	0.9900
C14—C15	1.377 (3)	C43—H43B	0.9900
			
C2—N1—C5	107.63 (10)	C13—C14—C15	123.04 (14)
C2—N1—C1	113.26 (10)	C14—C15—C16	118.06 (16)
C5—N1—C1	109.52 (10)	C14—C15—H15	121.0
C4—N2—C3	109.66 (10)	C16—C15—H15	121.0
C4—N2—C6	111.89 (10)	C15—C16—C11	120.84 (15)
C3—N2—C6	109.24 (11)	C15—C16—H16	119.6
C4—N2—H72	110.2 (13)	C11—C16—H16	119.6
C3—N2—H72	107.3 (13)	C22—C21—C26	118.88 (12)
C6—N2—H72	108.5 (13)	C22—C21—C1	121.80 (12)
N1—C1—C11	112.18 (11)	C26—C21—C1	119.25 (12)
N1—C1—C21	110.04 (10)	C21—C22—C23	120.90 (13)
C11—C1—C21	110.39 (11)	C21—C22—H22	119.6
N1—C1—H1	108.0	C23—C22—H22	119.6
C11—C1—H1	108.0	C24—C23—C22	117.97 (14)
C21—C1—H1	108.0	C24—C23—H23	121.0
N1—C2—C3	109.77 (10)	C22—C23—H23	121.0
N1—C2—H2A	109.7	F2—C24—C23	118.61 (15)
C3—C2—H2A	109.7	F2—C24—C25	118.12 (14)
N1—C2—H2B	109.7	C23—C24—C25	123.26 (13)
C3—C2—H2B	109.7	C24—C25—C26	117.53 (14)
H2A—C2—H2B	108.2	C24—C25—H25	121.2
N2—C3—C2	111.13 (11)	C26—C25—H25	121.2
N2—C3—H3A	109.4	C21—C26—C25	121.46 (14)
C2—C3—H3A	109.4	C21—C26—H26	119.3
N2—C3—H3B	109.4	C25—C26—H26	119.3
C2—C3—H3B	109.4	C36—C31—C32	118.35 (14)
H3A—C3—H3B	108.0	C36—C31—C8	118.57 (14)
N2—C4—C5	110.36 (10)	C32—C31—C8	123.04 (13)
N2—C4—H4A	109.6	C33—C32—C31	120.47 (15)
C5—C4—H4A	109.6	C33—C32—H32	119.8
N2—C4—H4B	109.6	C31—C32—H32	119.8
C5—C4—H4B	109.6	C32—C33—C34	120.35 (17)
H4A—C4—H4B	108.1	C32—C33—H33	119.8
N1—C5—C4	110.82 (11)	C34—C33—H33	119.8
N1—C5—H5A	109.5	C35—C34—C33	119.75 (16)
C4—C5—H5A	109.5	C35—C34—H34	120.1
N1—C5—H5B	109.5	C33—C34—H34	120.1
C4—C5—H5B	109.5	C34—C35—C36	119.97 (16)
H5A—C5—H5B	108.1	C34—C35—H35	120.0
C7—C6—N2	111.81 (12)	C36—C35—H35	120.0
C7—C6—H6A	109.3	C35—C36—C31	121.09 (16)
N2—C6—H6A	109.3	C35—C36—H36	119.5
C7—C6—H6B	109.3	C31—C36—H36	119.5
N2—C6—H6B	109.3	C44—O3—H3	109.5
H6A—C6—H6B	107.9	O2—C41—O1	123.88 (13)
C8—C7—C6	121.77 (14)	O2—C41—C42	118.34 (12)
C8—C7—H7	119.1	O1—C41—C42	117.73 (12)
C6—C7—H7	119.1	C43—C42—C41	115.51 (11)
C7—C8—C31	127.63 (14)	C43—C42—H42A	108.4
C7—C8—H8	116.2	C41—C42—H42A	108.4
C31—C8—H8	116.2	C43—C42—H42B	108.4
C12—C11—C16	118.89 (13)	C41—C42—H42B	108.4
C12—C11—C1	119.60 (13)	H42A—C42—H42B	107.5
C16—C11—C1	121.51 (12)	C44—C43—C42	115.52 (12)
C13—C12—C11	120.76 (15)	C44—C43—H43A	108.4
C13—C12—H12	119.6	C42—C43—H43A	108.4
C11—C12—H12	119.6	C44—C43—H43B	108.4
C14—C13—C12	118.41 (15)	C42—C43—H43B	108.4
C14—C13—H13	120.8	H43A—C43—H43B	107.5
C12—C13—H13	120.8	O4—C44—O3	122.28 (15)
F1—C14—C13	119.03 (17)	O4—C44—C43	123.32 (15)
F1—C14—C15	117.93 (18)	O3—C44—C43	114.37 (13)
			
C2—N1—C1—C11	45.82 (15)	C1—C11—C16—C15	179.64 (15)
C5—N1—C1—C11	165.97 (11)	N1—C1—C21—C22	−32.40 (17)
C2—N1—C1—C21	169.14 (11)	C11—C1—C21—C22	91.95 (15)
C5—N1—C1—C21	−70.72 (13)	N1—C1—C21—C26	150.61 (12)
C5—N1—C2—C3	62.13 (13)	C11—C1—C21—C26	−85.03 (15)
C1—N1—C2—C3	−176.65 (11)	C26—C21—C22—C23	0.0 (2)
C4—N2—C3—C2	54.54 (14)	C1—C21—C22—C23	−177.01 (12)
C6—N2—C3—C2	177.49 (11)	C21—C22—C23—C24	−0.1 (2)
N1—C2—C3—N2	−59.43 (14)	C22—C23—C24—F2	−177.93 (13)
C3—N2—C4—C5	−54.17 (14)	C22—C23—C24—C25	0.7 (2)
C6—N2—C4—C5	−175.54 (11)	F2—C24—C25—C26	177.40 (13)
C2—N1—C5—C4	−62.90 (13)	C23—C24—C25—C26	−1.3 (2)
C1—N1—C5—C4	173.58 (10)	C22—C21—C26—C25	−0.5 (2)
N2—C4—C5—N1	59.60 (14)	C1—C21—C26—C25	176.52 (13)
C4—N2—C6—C7	−55.86 (15)	C24—C25—C26—C21	1.2 (2)
C3—N2—C6—C7	−177.48 (11)	C7—C8—C31—C36	−179.03 (16)
N2—C6—C7—C8	115.58 (16)	C7—C8—C31—C32	−1.4 (2)
C6—C7—C8—C31	178.37 (14)	C36—C31—C32—C33	0.9 (2)
N1—C1—C11—C12	−111.85 (14)	C8—C31—C32—C33	−176.78 (15)
C21—C1—C11—C12	125.03 (13)	C31—C32—C33—C34	0.4 (3)
N1—C1—C11—C16	68.57 (17)	C32—C33—C34—C35	−1.2 (3)
C21—C1—C11—C16	−54.55 (17)	C33—C34—C35—C36	0.7 (3)
C16—C11—C12—C13	0.0 (2)	C34—C35—C36—C31	0.6 (3)
C1—C11—C12—C13	−179.58 (13)	C32—C31—C36—C35	−1.4 (2)
C11—C12—C13—C14	0.0 (2)	C8—C31—C36—C35	176.36 (15)
C12—C13—C14—F1	179.71 (16)	O2—C41—C42—C43	−169.74 (14)
C12—C13—C14—C15	−0.1 (3)	O1—C41—C42—C43	12.88 (19)
F1—C14—C15—C16	−179.64 (16)	C41—C42—C43—C44	177.78 (13)
C13—C14—C15—C16	0.1 (3)	C42—C43—C44—O4	161.8 (2)
C14—C15—C16—C11	−0.1 (3)	C42—C43—C44—O3	−20.2 (2)
C12—C11—C16—C15	0.1 (2)		

### Hydrogen-bond geometry (Å, º)

**Table:**

*D*—H···*A*	*D*—H	H···*A*	*D*···*A*	*D*—H···*A*
N2—H72···O1	0.96 (2)	1.73 (2)	2.6795 (16)	174 (2)
O3—H3···O1^i^	0.84	1.84	2.6564 (16)	162
C4—H4*A*···O4^ii^	0.99	2.58	3.287 (3)	128
C4—H4*B*···O2^iii^	0.99	2.39	3.3167 (19)	155
C25—H25···O2^iv^	0.95	2.53	3.4168 (19)	155
C12—H12···*Cg*^v^	0.95	2.81	3.7511 (17)	170

Symmetry codes: (i) *x*, −*y*+2, *z*+1/2; (ii) *x*, −*y*+2, *z*−1/2; (iii) *x*, −*y*+1, *z*−1/2; (iv) *x*+1, −*y*+1, *z*−1/2; (v) *x*, −*y*+1, *z*+1/2.
